# Sir Richard Quain

**Published:** 1891-04

**Authors:** 


### Sir Richard Quain.

Sir Richard Quain, the new medical baronet, is one of the members of the medical profession who is most popular outside his profession. For many years he has been *persona grata* in literary, artistic and official circles, and has numbered among his friends or acquaintances most of the great artists and men of letters of the last two generations. For in spite of his iron-gray hair, active habits and untiring energy in the discharge both of his professional and social obligations, Dr. Richard Quain must have already exceeded the Psalmist's span. At Mallow, Sir Richard first saw the light, and his first introduction to medical practice took place in

Limerick. He soon found his way to London and quickly began to make his mark, being for some years the righthand man of the late Dr. C. J. B. Williams, and then one of the physicians appointed at the institution of the Hospital for Consumptives at Brompton. For many years now he has possessed one of the leading consulting practices in London. His *magnum opus* is the famous "Dictionary of Medicine," which took some ten or twelve years to prepare, and in the production of which he obtained the assistance of most of the best known medical writers.—*London letter to Jour. Am. Med. Ass'n.*

